# Supplementary material for: Linear/Ladder-Like Polysiloxane Block Copolymers with Methyl-, Trifluoropropyl- and Phenyl- Siloxane Units for Surface Modification
Source: Polymers (Basel). 2021 Jun 23;13(13):2063. doi: 10.3390/polym13132063 (PMC8271457; doi:10.3390/polym13132063)
Supplement: Supplementary file 1 [file polymers-13-02063-s001.zip › polymers-1272356-supplementary.pdf]

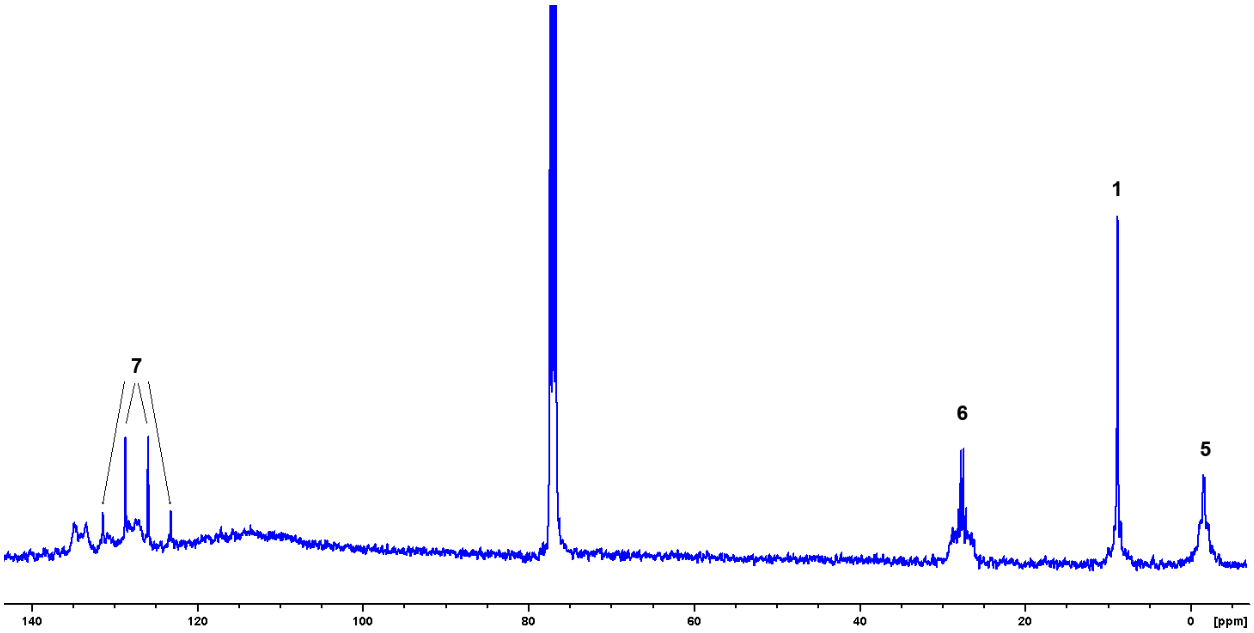

Figure S1. ^{13}C NMR spectrum of copolymer V in $CDCl_3$ solution.

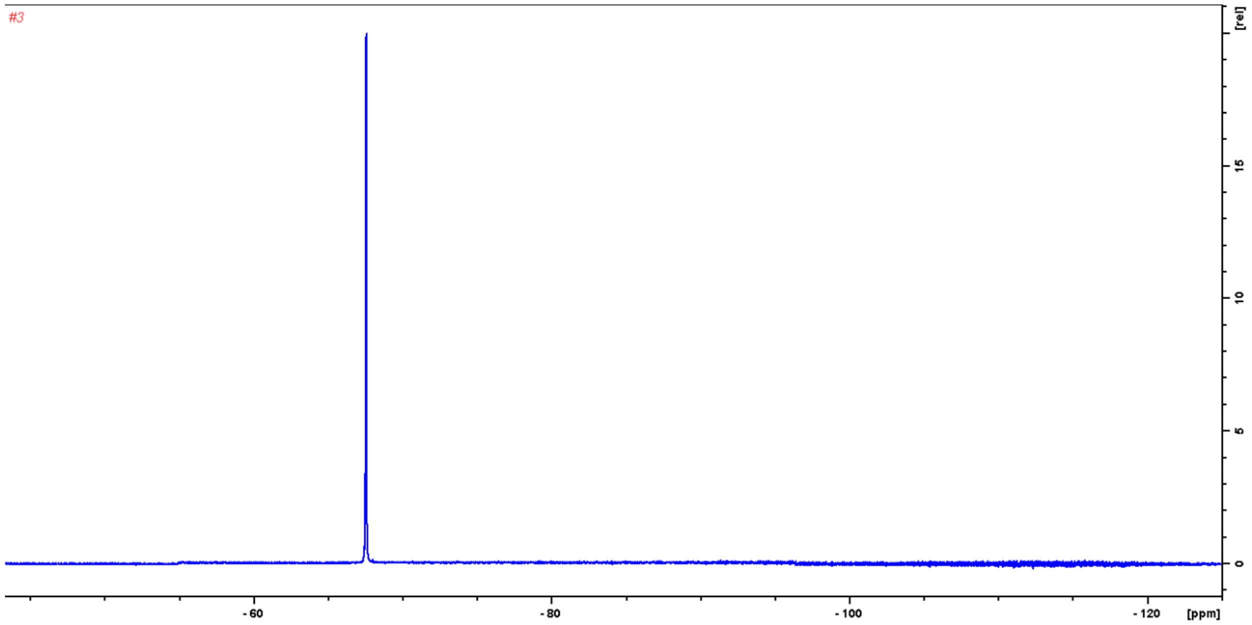

Figure S2. ^{19}F NMR spectrum of copolymer V in $CDCl_3$ solution.

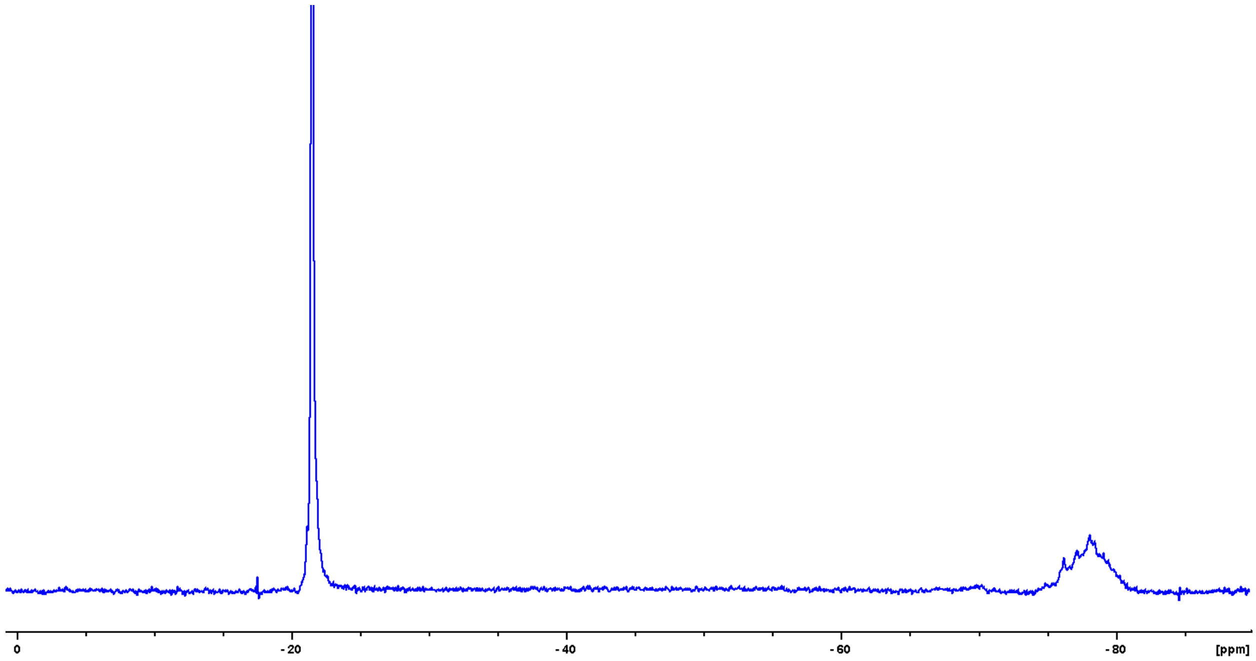

Figure S3. ^{29}Si NMR spectrum of copolymer IV in $CDCl_3$ solution.

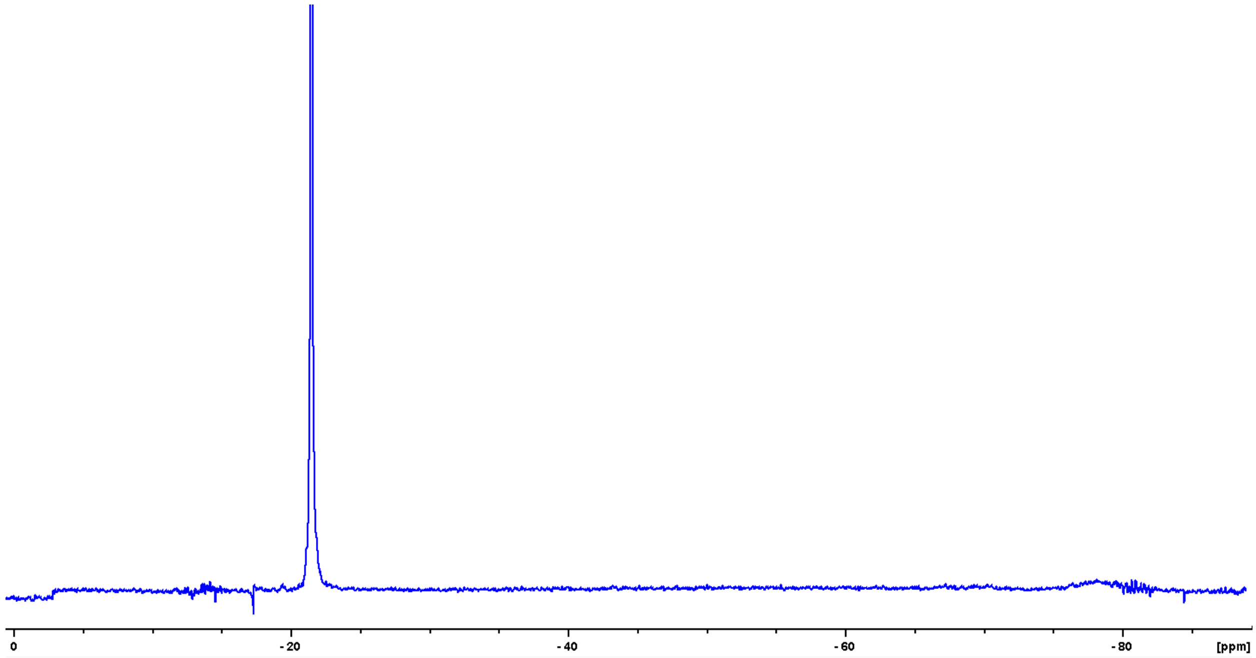

Figure S4. ^{29}Si NMR spectrum of copolymer V in $CDCl_3$ solution.
